# Apneic Technique in Laryngotracheal Surgery

**DOI:** 10.7759/cureus.21584

**Published:** 2022-01-25

**Authors:** Amy L Rutt, Klaus D Torp, Terrance Zimmermann, Paul Warner, Roger Hofer, Jonathan E Charnin, Dale Ekbom

**Affiliations:** 1 Otolaryngology, Mayo Clinic Alix School of Medicine, Jacksonville, USA; 2 Anesthesiology and Perioperative Medicine, Mayo Clinic, Jacksonville, USA; 3 Otolaryngology, Grand Strand Health, Myrtle Beach, USA; 4 Anesthesiology, Mayo Clinic, Rochester, USA; 5 Otolaryngology, Mayo Clinic, Rochester, USA

**Keywords:** airway mangement, thyroplasty, voice disorder, laryngotracheal surgery, apneic oxygenation

## Abstract

Background

Apneic oxygenation can be applied to select laryngotracheal procedures to improve operative visualization and avoid potential complications associated with intubation and jet ventilation.

Aims/objectives

The authors sought to determine if apneic oxygenation using a high-flow nasal cannula could be used as a safe alternative airway management strategy for the duration of select laryngotracheal procedures.

Methods

Single institution, multi-site retrospective review of 38 adult (>18 years old) patients undergoing apneic oxygenation in the setting of various laryngotracheal procedures from January 2017 through January 2018. Humidified oxygen was delivered via a high-flow nasal cannula. The data was collected and analyzed using SAS version 9.4 (SAS Institute, Cary, NC).

Results

Twenty-four women and 14 men, mean age 60.0 years (SD 16.1; 36-89) and 70.1 years (SD 7.2; 56-81), respectively, underwent a mean total apneic time of 23.9 minutes (13-40). A statistically significant correlation existed between apneic time and minimum oxygen saturation (Pearson correlation coefficient 0.38; p=0.018). Twenty-one patients resumed spontaneous ventilation without the need for jet ventilation, mask ventilation, or placement of a definitive airway during the procedure.

Conclusions and significance

Apneic oxygenation allows for extended periods of operating without the need for the placement of an endotracheal tube in patients undergoing general anesthesia for select laryngotracheal procedures.

## Introduction

A primary goal during general anesthesia is the maintenance of adequate oxygenation. After the induction of general anesthesia, while establishing an airway, there is a period of time known as the “apneic window” during which the patient is completely reliant upon the oxygen stores in the body - usually the functional residual capacity (FRC). The safe apneic window refers to the duration of time between ablation of patient respiratory effort and critical oxygen desaturation of approximately 88-90%, at which point further decreases in arterial oxygen tension (PaO2) lead to a rapid decline in oxygen saturation (SaO2) [1].

The primary technique to accomplish an extended apneic window is through ample preoxygenation of the awake patient prior to induction of general anesthesia, typically via the use of a face mask delivering 100% oxygen [2]. In an otherwise healthy preoxygenated adult patient, the apneic window may be as long as roughly ten minutes, compared to less than one minute without preoxygenation [1]. With the continued delivery of oxygen during apnea, including via high-flow nasal cannula, a pharyngeal oxygen reservoir can be established, which will prolong safe apnea time even further [3]. The role of this high-flow nasal cannula technique as a primary means of maintaining adequate hemoglobin saturation, as opposed to a bridge to the establishment of a definitive airway, has not yet been reported in the United States.

We sought to demonstrate that a safe and established method of providing apneic oxygenation could be used specifically in select laryngotracheal procedures to not only facilitate an improved operative experience without an obstructed view by endotracheal intubation but also to avoid potential risks associated with jet ventilation or establishment of a definitive airway. Here we report on our initial experience with transnasal, humidified oxygen to extend the apneic window, often for the entire duration of surgery, in select laryngotracheal procedures. 

## Materials and methods

The study was approved by the Mayo Clinic Institutional Review Board, and written informed patient consent was obtained on all subjects. The authors adhered to applicable Strengthening the Reporting of Observational Studies in Epidemiology (STROBE) guidelines for reporting research.

This was a single institution, multi-site retrospective review from January 2017 until January of 2018 of 38 adult patients undergoing apneic oxygenation in the setting of various laryngotracheal procedures, including vocal fold injections, resection of glottic and supraglottic lesions, and laser division of subglottic stenosis. Patients with known upper airway obstructive lesions that would preclude passage of oxygen between the pharynx and trachea or any laryngotracheal lesions with a high likelihood of intraoperative bleeding were excluded. Similarly, any patients with a history of skull base fracture, pneumocephalus, or elevated intracranial hypertension were excluded from undergoing apneic oxygenation via high-flow nasal cannula. Information about patients' age, sex, burden of medical co-morbidities (quantified using the Charlson co-morbidity score), and BMI were recorded. Premature cessation of apneic oxygenation, other means of oxygenation, and ventilation provided intraoperatively, and surgical procedures were also recorded. Maximum heart rate, blood pressure, minimum oxygen saturation (SpO2), and end-tidal CO2 level measured at the beginning and end of apnea were also noted.

Apneic time referred to the total time between cessation of spontaneous patient respiratory effort and end of the surgical procedure unless there was a need to commence ventilation due to less than 88% peripheral SaO2 via jet ventilation, bag-valve-mask, or endotracheal intubation. Optiflow™ (Fisher & Paykel, Auckland, New Zealand) high-flow nasal cannula system was the principal oxygen delivery device.

Patients were positioned on the operating table, in a head-elevated position of 20-30 degrees, with an Optiflow nasal cannula at 55-70 L/min delivering high-flow, warmed, and humidified 100% oxygen to the patient during spontaneous respiration for approximately ten minutes. General anesthesia was induced and maintained by a total intravenous route with neuromuscular blockade during the surgery. High-flow, 100% oxygen delivery continued after cessation of patient spontaneous respiratory effort. Initial end-tidal CO2 level was obtained by bag-valve-mask ventilation. This was recorded at the start of apnea by delivering approximately five breaths with positive pressure by mask. The high-flow nasal cannula was briefly removed during this interval (<30 seconds) to establish a tight seal between the patient's face and the mask, to record an accurate starting end-tidal CO2. Optiflow was then recommenced. The patient's angle of inclination was reduced to 0 degrees for direct, rigid laryngoscopy by the surgeon. During periods without the maintenance of rigid laryngoscopy, jaw thrust and neck extension were applied to maintain airway patency during apnea. Direct rigid laryngoscopy was performed using a Dedo, Ossoff-Pilling, Sataloff, or Lindholm laryngoscope, depending on the type of exposure best suited to the procedure.

High-flow oxygen was continued at 55-70 L/min until the end of the surgery, at which point neuromuscular blockade was reversed. At the end of apnea, either when a definitive airway was placed, or the procedure was completed, and the final end-tidal CO2 was recorded. In the case of CO2 or potassium titanyl phosphate (KTP) laser use, the delivery of high-flow oxygen via the Optiflow was reduced to 30-50% to mitigate the risk of airway fire despite the absence of flammable material in the airway. This would lead to a drop in SpO2, which then required establishing an alternative airway. Patients awakened in the operating room and were discharged home the same day of the procedure. There were no perioperative complications precluding patient same-day discharge. 

Continuous features were summarized with means, standard deviations (SDs), and ranges if approximately normally distributed and medians, interquartile ranges, and ranges otherwise. Categorical features were summarized with frequency counts and percentages. Associations of interest were evaluated using Pearson correlation coefficients and analysis of variance. Statistical analyses were performed using SAS version 9.4 (SAS Institute, Cary, NC). All tests were two-sided, and s p-value of <0.05 was considered statistically significant.

## Results

A summary of features collected for the 38 patients studied is shown in Table 1.

**Table 1:** Summary of features for patients who underwent apneic technique, N=38

Feature	Mean (SD; range)
Age in years	63.7 (14.3; 36-89)
Charlson index	2.3 (2.0; 0-6)
Body mass index	27.2 (5.0; 17.6-37.5)
Preoxygenation time using Optiflow	13.7 (5.5; 5-28)
Starting end-tidal CO_2_ (N=26)	32.1 (4.7; 20-41)
Ending end-tidal CO_2_	54.4 (11.9; 20-81)
Apneic time	23.9 (6.1; 13-40)
Rate of CO_2_ increase (N=26)	1.12 (0.46; 0.38-2.56)
Minimum oxygen saturation	93.6 (6.1; 79-100)
Maximum systolic blood pressure	166 (35; 114-237)
Minimum systolic blood pressure	98 (21; 68-166)
Sex	N (%)
Female	24 (63)
Male	14 (37)
Mallampati grade (N=37)	
1	13 (35)
2	14 (38)
3	9 (24)
4	1 (3)

Sample sizes for features with missing data are indicated in parentheses. The mean total apneic time was 23.9 minutes (range 13-40). The mean rate of rise of end-tidal CO2 (mmHg/min) was 1.12 (range 0.38-2.56), and the median total procedure time was 21.5 minutes (interquartile range 17-59; range 7-119).

Associations of interest with apneic time and rate of CO2 increase are shown in Tables 2 and 3, respectively.

**Table 2:** Summary of associations with apneic time, N=38

Feature	Correlation		P-value	
Age in years	0.14		0.14	
Charlson index	0.11		0.51	
Body mass index	-0.24		0.15	
Preoxygenation time using Optiflow	-0.03		0.86	
Starting end-tidal CO_2_ (N=26)	0.43		0.029	
Ending end-tidal CO_2_	0.18		0.28	
Minimum oxygen saturation	0.39		0.018	
Maximum systolic blood pressure	0.20		0.23	
Minimum systolic blood pressure	0.09		0.58	
Sex	Mean (SD, range)			
Female	24.1 (6.3; 13-40)			
Male	23.5 (6.0; 16-35)			
Mallampati grade (N=37)				
1	22.7 (5.6; 14-33)			
2	23.1 (5.4; 13-33)			
3 or 4	26.3 (7.8; 16-40)			

**Table 3:** Summary of associations with the rate of CO2 increase, N=26

Feature	Correlation	P-value
Age in years	0.16	0.45
Charlson index	0.14	0.49
Body mass index	0.13	0.52
Preoxygenation time using Optiflow	-0.04	0.86
Minimum oxygen saturation	-0.18	0.38
Maximum systolic blood pressure	-0.03	0.9
Minimum systolic blood pressure	-0.09	0.65
Sex	Mean (SD; range)	
Female	1.02 (0.33; 0.38-1.44)	0.23
Male	1.25 (0.59; 0.52-2.56)	0.21
Mallampati grade (N=25)		
1	1.29 (0.54; 0.52-2.56)	
2	1.18 (0.23; 0.87-1.41)	
3 or 4	0.92 (0.44; 0.38-1.43)	

There was a statistically significant correlation between apneic time and minimum SaO2 recorded during the surgery (Pearson correlation coefficient 0.38; p=0.018), indicating that lower SaO2 correlated with shorter apneic times. There was also a statistically significant positive correlation between apneic time and starting end-tidal CO2 (Pearson correlation coefficient 0.43; p=0.029).

Twenty patients underwent vocal cord injections using either adipose or micronized acellular dermis; nine patients underwent excision of glottic pathology with or without the use of KTP or CO2 laser; six patients underwent treatment for idiopathic subglottic stenosis using CO2 laser; two patients underwent KTP laser ablation for treatment of recurrent respiratory papillomatosis; and one patient underwent removal of a supraglottic lesion. None of the patients had acute or delayed airway compromise or postoperative stridor after treatment.

Twenty-one of the 38 patients (55.3%) resumed spontaneous ventilation without the need for the placement of an alternative airway following the surgical procedure. Thirteen patients (34.2%) required intubation with a cuffed endotracheal tube prior to the end of the procedure due to SpO2 falling below 88%. Four patients (10.5%) were initiated on jet ventilation prior to the end of the procedure in order to complete treatment of their subglottic stenosis. In nine of the 17 patients requiring an alternative airway, laser and the reduction in the fraction of inspired oxygen (FiO2) led to the alternative airway management.

## Discussion

The physiology of apneic oxygenation is based on the passive exchange of CO2 and oxygen at the level of the alveoli in the absence of ventilation. While apneic oxygenation can maintain oxygen saturations near 100% even after 100 minutes of apnea under ideal conditions [4], there is no mechanism other than the blood’s buffering system to counteract the rise of CO2. The factors that moderate the rate of rise of CO2 center both on preoxygenation strategies [5] and the continuous insufflation of the airway with high-flow oxygen.

A nasal cannula can be used safely and efficaciously for continuous delivery of oxygen during apnea. The pharynx will continue to function as an oxygen reservoir, even when the mouth is open, provided a patent passageway is maintained between the pharynx and alveoli, markedly extending the apneic window. Due to the differential passive rates of oxygen and CO2 exchange at the level of the alveoli during apnea, a negative pressure gradient of up to 20 cm H2O induces oxygen diffusion across the alveolar membrane despite an apparent lack of mechanical ventilation [6].

Despite adequate oxygenation via this method, without placement of a definitive airway or other means of ventilation, the obvious consideration involves the resultant hypercapnia and the potential for acidosis associated with aventilatory mass flow (AVMF). On average, partial pressure of carbon dioxide (PaCO2) increases 8 to 16 mmHg in the first minute of apnea and then approximately 3 mmHg/minute thereafter in apneic patients without high-flow oxygen [7]. This degree of hypercarbia during short durations is typically not clinically significant, except in the rare cases of pre-existing profound metabolic acidosis, which can lead to cardiovascular collapse [8] predominantly due to the development of an unstable cardiac arrhythmia. Concomitant increased intracranial pressure must also be a consideration, where further increases in PaCO2 can lead to detrimental cerebral vasodilation [1].

In our study, there were no adverse cardiopulmonary events during any of the cases, and no ST-segment changes >2 mm in the V5 lead occurred. There were no postoperative complications.

When preoxygenating any patient prior to apnea, positioning plays a major role, as supine positioning predisposes the lungs to atelectasis and facilitates soft tissue collapse in the upper airway [9]. A significant increase in time to desaturation following apnea has been observed in patients positioned with a 20-degree head-up position versus supine positioning [10-13].

Duration of preoxygenation is another key factor. In our study, we preoxygenated patients for a mean time of 13.7 minutes using high-flow nasal oxygen, which in addition to creating positive pressure to help prevent atelectasis, also minimized soft upper airway collapse.

Apneic oxygenation has been explored in the operative setting by several authors [6]. In 2014, Patel et al. performed a landmark study named “Transnasal Humidified Rapid-Insufflation Ventilatory Exchange (THRIVE),” evaluating 25 patients with difficult airways who underwent general anesthesia for hypopharyngeal or laryngotracheal surgery [6]. Patients had continuous delivery of transnasal high-flow humidified oxygen from the preoxygenation period throughout neuromuscular blockade until a definitive airway was secured. The median apnea time was 14 (5-65) minutes, with no patients experiencing arterial desaturations <90% [6]. In two of the 25 patients in the THRIVE study, apneic oxygenation using the high-flow nasal cannula was used throughout the entirety of the procedure without placement of a definitive airway, with apneic times of 32 and 65 minutes, respectively [6].

In our study, we were able to obtain average apneic times of 23.9 minutes, with 21 of 38 patients (55.3%) utilizing apneic oxygenation for the entirety of their procedure. Nine of the 17 patients requiring placement of a definitive airway underwent procedures involving CO2 or KTP laser and thus had decreased FiO2 during the procedure. The average rate of rise of end-tidal CO2 was 1.12 mmHg/min, which compared favorably to the rates of increase demonstrated by other authors [6,11,14]. Furthermore, we found that lower SaO2 correlated positively with shorter apneic times, which was to be expected since once the oxygen saturations drop, they are difficult to treat with apneic oxygenation alone. A positive correlation was found between apneic time and starting end-tidal CO2, indicating that higher starting end-tidal CO2 values were associated with longer apneic times. This physiologic basis for this finding was unclear.

Several patient factors must be considered when implementing apneic oxygenation during general anesthesia for surgical procedures. Increased BMI, high Mallampati class, retrognathia, cardiopulmonary disease, increased Cormack-Lehane grade, and upper airway obstruction from neoplastic causes can all theoretically negatively impact direct laryngoscopy. If those factors affect an open airway passage, the delivery of oxygen via a high-flow nasal cannula can be affected, leading to a shorter safe apnea time. It is important to consider in critically ill patients with poor cardiac output, ventilation-perfusion mismatch, and increased metabolic demands that oxygen storage in the lungs may be depleted at a much faster rate at the beginning of apnea. The choice of paralytic agent given may also influence apneic time, with patients receiving rocuronium experiencing significantly longer times to desaturation <95% compared to patients receiving succinylcholine, perhaps due to increased oxygen extraction secondary to succinylcholine-induced muscle fasciculations [15-17]. 

Apneic oxygenation can be a helpful tool either as a sole airway management strategy or as an adjunct to prolong safe apnea time until a definitive airway can be established. Future prospective studies will be useful to compare the best method for monitoring tissue CO2 concentrations (transcutaneous vs. arterial vs. end-tidal), as well as to elucidate which patient characteristics might lead to shortened apneic times (morbidly obese vs. obstructive sleep apnea, for instance).

Criticisms of this study may include the retrospective nature and lack of standardized data collection across sites. Another potential limitation of the study was the measurement technique for the collection of end-tidal CO2. Measurement of end-tidal CO2 using a mask could allow for leakage of air and thus carry the potential for measurement error. It should be noted that in order to obtain starting end-tidal CO2 readings, approximately five positive-pressure breaths by hand ventilation with a mask were delivered. Although apneic time recording was started at the initiation of neuromuscular blockade, these few breaths prior to the initiation of the procedure did represent a brief interruption in the aventilatory period.

## Conclusions

In our study, we demonstrated that high-flow oxygen nasal cannula oxygenation was beneficial in extending the apneic period in patients undergoing general anesthesia for laryngotracheal procedures. The mean minimum oxygen saturation was 94%, with a mean apneic time of 24 minutes. No patients developed adverse cardiopulmonary events perioperatively, and there was no clinical indication of respiratory acidosis. Given these findings, it seems appropriate to use this oxygenation technique for select patients requiring laryngotracheal surgery. This study is important because it demonstrates that this method is a safe and efficacious airway adjunct. A backup plan for ventilation should be agreed upon prior to the surgery by both the surgeon and anesthesia team. There are physiological benefits to both the upper and lower airways attributed to high-flow nasal cannula oxygenation. Future studies should examine the application of this technique in emergency situations, high BMI patients, and patients with more pronounced systemic disease. 
